# Crack cocaine users views regarding treatment with contingency management in Brazil

**DOI:** 10.1186/s13011-018-0144-7

**Published:** 2018-02-12

**Authors:** André Q. C. Miguel, Clarice S. Madruga, Viviane Simões, Rodolfo Yamauchi, Claudio J. da Silva, Renata R. Abdalla, Michael McDonell, Sterling McPherson, John M. Roll, Jair J. Mari, Ronaldo R. Laranjeira

**Affiliations:** 1 National Institute of Policies on Alcohol and Drugs (INPAD) of the Department Psychiatry and Medical Psychology of the Federal University of São Paulo (UNIFESP), São Paulo, Brazil; 2 Department of Psychiatry and Medical Psychology of the Federal University of São Paulo- UNIFESP, São Paulo, Brazil; 3 Program of Excellence in Addictions Research, Washington State University, Spokane, USA

**Keywords:** Crack cocaine, Contingency management, Behavioral treatment, Ambulatory treatment, Treatment acceptance, Brazil

## Abstract

**Background:**

Contingency management (CM) has recently shown efficacy in promoting abstinence and retention in treatment among crack cocaine users in Brazil. However, partially because of unawareness and resistance among health care providers, CM has not been widely employed. The objective of this study was to conduct a secondary analysis in order to evaluate how CM participants perceive their treatment experience.

**Methods:**

Twenty-seven crack cocaine users, previously assigned to 12 weeks of CM treatment, were assessed with a structured questionnaire designed to assess their personal opinion of, difficulty in understanding, and acceptance of the CM intervention, as well as their opinion regarding its impact on their treatment responses.

**Results:**

Descriptive analyses showed that 92.6% of the participants found it very easy to understand the CM protocol. All participants reported liking their CM experience quite a bit. For the perceived effects of CM on their treatment response, 81.5% of the participants stated that CM helped them considerably, the mean score for the impact of CM on treatment response (out of a maximum of 10) being 9 (SD = 1.5). When asked if they believed CM could help other people with crack cocaine dependence, 92.6% of the participants stated that CM could help such people a lot and 7.4% stated that it could help them a little.

**Conclusions:**

From the perspective of the patients, CM was easily assimilated, easily accepted, and had a direct positive effect on treatment response. These findings provide additional support for the incorporation of CM into substance abuse treatment services in Brazil.

## Background

In the last 20 years, the demand for crack cocaine dependence treatment has increased drastically in Brazil, and crack cocaine use has become a severe health concern in the country [1, 2]. In Brazil, crack cocaine use is associated with high rates of psychiatric comorbidities, cognitive impairment, unemployment, homelessness, sexually transmitted infections, involvement in illegal activities, incarceration, and death [3–10]. When compared to snorted cocaine users, crack cocaine users present poor treatment outcomes [11], with high dropout rates [6] and low post-treatment abstinence rates [12].

The use of Contingency Management (CM) to treat substance use disorders has been studied in the United States [13–15], Spain [16–18], the United Kingdom [19] and China [20] over the last 30 years. CM interventions are based on operant behavior principles that acknowledge that a specific response will have a greater likelihood of occurring if it is immediately followed by a reinforcing consequence [21, 22]. Under this framework, the CM interventions deliver systematic rewards (such as vouchers with a monetary value) contingent upon specific desirable responses (such as objective verification of abstinence) [23, 24].

A recent randomized clinical trial conducted in the city of São Paulo, Brazil, presented strong evidence that CM is an effective treatment for crack cocaine dependence [25]. In that trial, CM was significantly more efficacious than was usual care in increasing rates of treatment session attendance and retention in treatment, as well as in reducing crack cocaine use and promoting continuous abstinence from crack cocaine. The authors also observed significant secondary effects of CM, including reductions in alcohol and marijuana use, as well as in post-treatment anxiety and depressive symptomatology [25, 26].

Although CM shows promise as an effective intervention to address the high morbidity and mortality associated with crack cocaine use, health providers at substance abuse treatment facilities are unaware of the positive effects of CM on treatment responses. Given the evidence of strong resistance to the use of CM among regular treatment staff, as shown in studies conducted in the United States [27, 28], it is important to provide health professionals with additional information regarding the benefits of incorporating CM into routine substance abuse treatment protocols [29]. One strategy to accomplish this is to provide crack cocaine users with the perspectives of patients regarding their experience with the CM intervention. Therefore, the aim of this study was to access how crack cocaine users exposed to a CM intervention in Brazil evaluated their experience with this novel treatment intervention.

## Methods

### Design

For this study, we conducted secondary analyses of data collected in a randomized clinical trial developed between August 2012 through to July 2015, which was designed to evaluate the efficacy of CM in improving attendance and retention in treatment, as well as in reducing crack cocaine use and promoting continuous crack cocaine abstinence, in a sample of crack cocaine-dependent individuals seeking treatment at the Vila Maria Specialized Medical Outpatient Clinic for Alcohol and Drug Treatment, located in the northern region of the city of São Paulo [25]. Treatment at Vila Maria Outpatient clinic consisted of 90 min group therapy sessions a week that were focused on coping skills, training and relapse prevention; 90 min group occupational therapy weekly sessions; at least one individual session per month with a psychiatrist; and one individual psychotherapy session per week.

### Participants

Individuals between 18 and 60 years of age, seeking treatment for crack cocaine dependence, as defined by the current criteria of the Diagnostic and Statistical Manual of Mental Disorders, Fourth edition (DSM-IV), assessed with the Structured Clinical Interview for DSM-IV Axis I Disorders [30], were screened for eligibility. Polydrug users were included if crack cocaine was the drug of choice. The exclusion criteria were being abstinent from crack cocaine for 4 or more weeks, being diagnosed with schizophrenia (confirmed using the structured interview), and not being able to attend treatment sessions three times per week. A total of 65 individuals met the study criteria and were enrolled in the study. Of those 65 participants, 33 were allocated to receive the CM intervention and 32 were allocated to receive the usual care intervention. All participants provided written informed consent. The study was approved by the Research Ethics Committee of the Federal University of São Paulo and by the Ethics Committee from the Brazilian National Ministry of Health (CAAE No: 00745912.4.0000.5505).

### Procedure

**Fig. 1:**
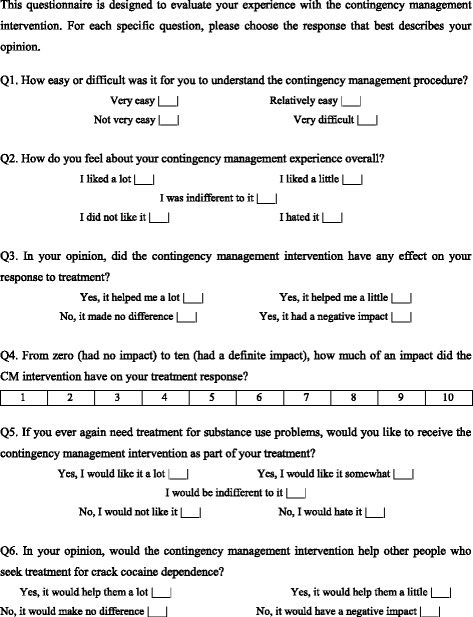
Contingency management treatment experience questionnaire

For 12 weeks, participants allocated to the CM intervention were encouraged to come to treatment sessions three times per week (Mondays, Wednesdays, and Fridays), at which time they would submit urine samples in order to identify crack cocaine use. Participants could earn vouchers with monetary value when they submitted samples testing negative for crack cocaine. Vouchers values increased when consecutive negative samples for crack cocaine were submitted but reset to the original value if participants missed a screening appointment or tested positive for crack cocaine. Vouchers could be used to obtain any goods available in the surrounding community, with the exception of tobacco and alcohol. If abstinent for all 12 weeks of the CM intervention, participants would receive a total of US$235.50 in vouchers. For a full description of the methods employed in the clinical trial, see Miguel et al. (2016). At the end of treatment, a total of 27 participants (81.8%) underwent post-treatment evaluation with a 6-item structured questionnaire designed to assess participant opinions regarding the ease or difficulty of understanding the CM protocol, acceptance of the CM intervention, and the impact of CM on treatment responses (Fig. 1).

For baseline variables, descriptive data analyses were conducted considering the entire sample allocated to the CM intervention (*n* = 33). For all variables of the 6-item structured questionnaire, descriptive data analyses were conducted considering only the participants interviewed in the post-treatment assessments (*n* = 27). All statistical analyses were performed with the IBM Statistics software package, version 22.0 (IBM Corporation, Armonk, NY, USA).

## Results

The sample was predominantly male (90%) with a mean age of 35.3 (SD = 8.7), low education achievement (mean years of education = 8.9; SD = 3.4) and high rates of unemployment (84.8%). Nearly a fifth (18.2%) were currently living in the streets at the time of baseline assessment. Regarding substance use disorders, 66.7% had multiple substance dependence, with high prevalence of concomitant alcohol dependence (63.6%). The presence of other psychiatric symptomatology was also prevalent, with nearly half of the sample (45.5%) presenting at least one psychotic symptom, 90.9% presenting at least mild anxiety symptomatology (accessed with de Beck Anxiety Inventory), and all of the participants presenting at least mild depressive symptomatology (accessed with de Beck Depression Inventory-II).

Regarding the difficulty in understanding the CM procedure, 92.6% of the participants found it very easy to understand, whereas 7.4% found it relatively easy to understand. The CM intervention was well accepted by the participants, all of whom responded, “I liked it a lot” to the question “How do you feel about your CM experience?” For the perceived effects of CM on treatment response, 81.5% of the participants reported that the CM treatment helped them “a lot” in achieving their treatment responses, whereas 8.5% reported that it helped them “a little”. The mean score for the impact of CM on treatment response reported by participants (out of a maximum of 10) was 9 (SD = 1.5). When asked if they would like to receive CM intervention as part of future treatments, all participants responded, “Yes, I would like it a lot”. Finally, 92.6% of the participants believe that the CM intervention could help other crack cocaine-dependent individuals, the remaining 7.4% believing that it could help such individuals at least a little.

## Discussion

Crack cocaine use has become a severe public health problem in Brazil, and treatment services are struggling to provide effective treatment for crack cocaine dependence. This is a scenario which enlaces the sheer severity of crack cocaine dependence, the extreme social vulnerability in which crack cocaine dependent individuals are living in Brazil, and the shortage of adequate treatment resources, which is seen through the lack of evidence of efficacy in public outpatient treatment services for this population [25]. Recent evidence of the efficacy of CM in treating crack cocaine dependence in the city of Sao Paulo suggests that CM is a viable approach to addressing this public health concern. However, treatment provider awareness and acceptance of CM continues to be an important issue that needs to be addressed in order to promote the introduction of CM into routine substance abuse treatment protocols in Brazil. In the United States, despite the robust evidence of CM’s efficacy in the treatment of substance use disorders, regular clinical staff continues to show resistance to this method of intervention [27, 28]. As a consequence, the diffusion of CM to clinical practice in American soil has been modest at best [31, 32]. In order to bridge-the-gap between science and practice, the National Institute of Drug Abuse (NIDA) has fostered the Clinical Trial Network initiative were CM randomized clinical trials have been developed in several outpatient treatment settings and in collaboration with regular treatment staff [33, 34]. These trials have not only demonstrated the effectiveness of CM when delivered in natural outpatient clinical settings [35, 36] but also suggest that exposing regular clinical staff to CM interventions is effective in promoting awareness, reducing resistance and prompting the dissemination of CM [37].

A further alternative to promote awareness of this type of intervention and reduce resistance by treatment providers is to present patients’ perspectives of their experience with a CM intervention. The present study provides additional knowledge of the impact of CM interventions in crack cocaine users in Brazil by evaluating how treatment-seeking crack cocaine users perceived their experience with the intervention. To our knowledge this was the first study to focus on the participants’ perspective regarding CM interventions. We found that the CM protocol was easily understood and well accepted by the participants. In addition, participants exposed to the CM condition felt that it had a direct positive effect on their treatment response and that it would be an effective approach to help other people who suffer from crack cocaine dependence in Brazil. These findings offer further support for the introduction of CM into routine substance abuse treatment protocols in Brazil. They suggest that CM interventions are easily understood, are well accepted, and have a direct positive impact on treatment response for treatment-seeking crack cocaine users in Brazil.

### Limitations

This study has several limitations that would need to be considered. First, our sample was small and all of the participants were recruited from the same treatment center. The results might have been different if a greater number of participants, from multiple treatment centers, had been included. Second, the data presented in this manuscript were obtained from only 81.5% of the sample exposed to the CM condition. Consequently, patients not retained until the post-treatment assessments, possibly with worse treatment responses, might have had a less favorable opinion of CM. It is important to highlight, however, that the Ethics Committee from the Brazilian National Ministry of Health prohibits the use of payment to collect data from human subjects at baseline, end-of-treatment and follow-up assessments. As a consequence, missing data at the end-of-treatment and follow-ups are usually higher than in countries were participants are paid to respond to end-of-treatment and follow-up assessments. As so, it is possible to assume that the percentage of missing data observed in this study was in part influenced by this factor.

Finally, it is important to consider that the majority of our sample had low income and were living in extremely poor social conditions. As a consequence, CM might have had a larger impact on their economic condition than in their crack cocaine dependence treatment per-se. The assessment did not differentiate these two outcomes and therefore was unable to determine whether, CM was more helpful by promoting periods of continuous abstinence or by improving their economic condition according to the participant’s perspectives. It could have been evaluated by including questions such as: “In your opinion, did the contingency management intervention help you achieve periods of abstinence?” and “Did contingency management intervention help you in your economical condition?” in the Contingency Management Treatment Experience Questionnaire; and they are suggested for future studies.

## Conclusions

Despite its limitations, our study provides important information on how crack users perceive the CM intervention. In a country were public treatment services for crack cocaine dependent individuals lack evidence of efficacy, the propagation of evidence based interventions is paramount. CM has recently shown efficacy in promoting retention in treatment, reducing crack cocaine use, promoting abstinence and also, reducing psychiatric symptomatology for treatment seeking crack cocaine dependent individuals in Sao Paulo, Brazil. This study has shown that, according to this population, CM was clearly and easily understood, well accepted and had a positive effect on their treatment response. Our results offer additional support for the applicability of CM in outpatient public treatment settings and advocates for the distribution of CM to other substance abuse treatment centers in Brazil.

## Abbreviations

CM: Contingency Management
DSM-IV: Diagnostic and Statistical Manual of Mental Disorders, Fourth edition
NIDA: National Institute of Drug Abuse
